# Implementation of stimuli with millisecond timing accuracy in online experiments

**DOI:** 10.1371/journal.pone.0235249

**Published:** 2020-07-10

**Authors:** Zhengguo Gao, Biao Chen, Tianwen Sun, Haoqiang Chen, Kai Wang, Peipei Xuan, Zhen Liang

**Affiliations:** 1 Department of Biomedical Engineering, School of Life Science, Anhui Medical University, Hefei, Anhui Province, People’s Republic of China; 2 Department of Computer Science, School of Humanistic Medicine, Anhui Medical University, Hefei, Anhui Province, People’s Republic of China; 3 Laboratory of Cognitive Neuropsychology, Department of Medical Psychology, Anhui Medical University, Hefei, Anhui Province, People’s Republic of China; 4 School of Foreign Languages, Anhui Agricultural University, Hefei, Anhui Province, People’s Republic of China; Wright State University, UNITED STATES

## Abstract

Online experiments are growing in popularity. This study aimed to determine the timing accuracy of web technologies and investigate whether they can be used to support high temporal precision psychology experiments. A dynamic sinusoidal grating and flashes were produced by setInterval, CSS3, and requestAnimationFrame (hereafter, rAF) technologies. They were run at normal or real-time priority processing in Chrome, Firefox, Edge, and Internet Explorer on Windows, macOS, and Linux. Timing accuracies were compared with that of Psychtoolbox which was chosen as gold standard. It was found that rAF with real-time priority had the best timing accuracy compared to the other web technologies and had a similar timing accuracy as Psychtoolbox in traditional experiments in most cases. However, rAF exhibited poor timing accuracy on Linux. Therefore, rAF can be used as technical basis for accuracy of millisecond timing sequences in online experiments, thereby benefiting the psychology field.

## Introduction

Internet technology has been becoming progressively more mature, and the experimental methods in many fields are being deeply affected. Online experiments are increasingly replacing traditional experiments as the advantages of online experiments are gradually recognized. There are many advantages to online experiments. For example, the experimental conditions are flexible and can be conducted at any time and any place with Internet access. Furthermore, subjects are not limited to a particular group of people [1]. Currently, there are many online software packages and platforms that have been developed; examples are jsPsych, and Lab.js, which are browser-based online experiment tools [2,3]. De Leeuw found that JavaScript, which is used in jsPsych, is an appropriate tool for measuring response time in online behavioral experiments [4].

However, online experiments are currently limited by immature technologies, among other factors. For example, not all experiments are easy to migrate to the Internet. Plant [5] suggested that the lack of millisecond accuracy can cause psychological experiments to not be repeatable. Schmidt similarly [6] suggested that the timing accuracy is too low to support the development of online experiments. However, van Steenbergen and Bocanegra argued that the timing accuracy of online experiments is acceptable [7]. In 2014, this issue was discussed on GitHub (https://github.com/jspsych/jsPsych/issues/75). Stian Reimers argued that the timing accuracy of rAF was much more consistent than that of standard timestamps. However, neither side of the debate provided the exact timing accuracy of the online experiment. Therefore, if we can accurately determine the timing accuracy of online experiments by external measurements, we will not only settle this dispute but also define the scope of online experiments.

This study aimed to examine the best possible timing accuracy of stimuli presented in browsers and corresponding approaches. There are two classes of methods that can render dynamic stimuli in a browser: one is based on plug-in components such as Adobe Flash [8], and the other is based on web technologies. Adobe Flash was not tested because it is being phased out, and most browsers do not automatically support Flash plug-ins. Four methods fall under web technologies: setInterval, rule keyframes of CSS3 (hereafter, CSS3), requestAnimationFrame, and Web Animation API. Web Animation API is a new technology that is still in a draft state, and most browsers are incompatible with it [9]; thus, it was not investigated in this article. For comparison with traditional experiments, the timing accuracy of Psychtoolbox [10] (hereafter, PTB) was measured as a gold standard.

## Materials and methods

Considering the potential inaccuracies that can arise when stimuli are presented on different browsers, operating systems, and computers, we developed an external measurement system to validate timing accuracy. A photosensitive triode was placed on computer screen and connected to a photoelectrical convertor (see S1 File). After photoelectrical conversion, the electrical signal was sent to a logic analyzer (Saleae logic 16, USA). Unless otherwise noted, the sampling rate of the logic analyzer was set to 6.25 MS/sec for digital signal and 1.563 MS/sec for the analog signal, providing sub-microsecond accuracy. Digital and analog signals were used for mutual authentication to confirm the reliability of the measurement system.

### Stimuli

Dynamic sinusoidal gratings and flashes are typical visual stimuli for psychological experiments. The luminance in the time dimension of dynamic sinusoidal gratings changes gradually, whereas that of flashes changes dramatically. Therefore, we used both stimuli to assess timing accuracy. The temporal characteristic of gratings was set at 16 frames/period and that of the flashes was 2 and 16 frames/period. The size of all stimuli was the same: 512 × 512 pixels. The code and procedure used to generate stimuli can be found in the S1 File. It is noted that the method of CSS3 animation is called “steps,” and is used to simulate the luminance changes in Psychtoolbox.

#### Browsers and computers

All measurements were performed on three different computers. The first computer (computer 1) was a desktop computer running Windows 10, with an Intel i7-4750 quad core processor, 8 GB RAM and an AMD Radeon R7 200 Series GPU. Experiments were conducted in Chrome 67, Firefox 59, Edge 42, and Internet Explorer 11 on an Acer V223HQL monitor running at 60 Hz. The second computer (computer 2) was a desktop computer running Windows 10, with an Intel I5-8500 six-core processor, 8 GB RAM and an NVIDIA GeForce GTX 1050 Ti GPU running Chrome 67, Firefox 59, Edge 42, and Internet Explorer 11 on an ROG PG279Q monitor running at 60 or 144 Hz. The web browsers were exactly the same as on the first computer. This computer also ran Linux (ubuntu 19.0.4) with Chrome 14 and Firefox 66. The third computer (computer 3) was an old MacBook Pro (15-inch, mid-2010), running macOS High Sierra 10.13.6 with Chrome 74, Firefox 66, and Safari 12.0 on an Intel Core i5 with 4 GB RAM and an Intel HD Graphics 288 MB GPU. The monitor ran at 60 Hz. Psychtoolbox (3.0.14) based on MATLAB (2010a) ran on the first computer.

#### Boosting priority

Priority is a key point for improving the timing accuracy of dynamic stimuli and can be automatically boosted by the priority function in PTB. However, it was impossible to boost priority using JavaScript in all browsers. Therefore, we boosted the priority manually by using the “renice priority pid” command in Linux, and by setting the priority in Task Manager in Windows. The priority of macOS browsers was set by default because macOS automatically downgrades or upgrades it (http://psychtoolbox.org/docs/Priority). In Windows, boosting priority was achieved by using “Windows Task Manager”. Once Task Manager is opened, we navigate to the “Processes” tab, right-click on the running browser, and change its priority using the “Set Priority” menu. In Linux, we open a “Terminal” window, and then type in “top” to get the pid of the browser. Finally, we type “renice -20 pid” and press the return key. Then the browser runs in real-time priority.

#### Data analysis

Offline analysis was performed in MATLAB (2010a), and figures generated using GraphPad Prism (6.0). The timestamp for when the signal first rose to the threshold was defined as the start time of a period and the end of the previous period. Periods were defined as the difference between adjacent timestamps. To clearly observe timing accuracies, we converted all durations of stimulus presentation from seconds to frames. The mean, standard deviation, range, and frame loss rate of the periods were calculated in MATLAB.

For convenience, longer-than-intended periods were called “longer periods” (the period was longer than the intended period by 0.1 frames); shorter-than-intended periods were called “shorter periods” (the period was 0.1 frames shorter than the intended period). Frame loss rate (hereafter, FLR) is defined as the proportion of longer and shorter periods among all periods.

## Results

### Experiment 1

We presented dynamic sinusoidal gratings in a Chrome browser on computer 1. Chrome’s priority was manually set to normal while PTB was boosted to the maximum level by the priority function. Dynamic sinusoidal gratings were generated using setInterval, CSS3, and rAF on a canvas in HTML5. The period of dynamic sinusoidal gratings was set to 16 frames. (the procedures can be found in the S1 File).

**Fig 1:**
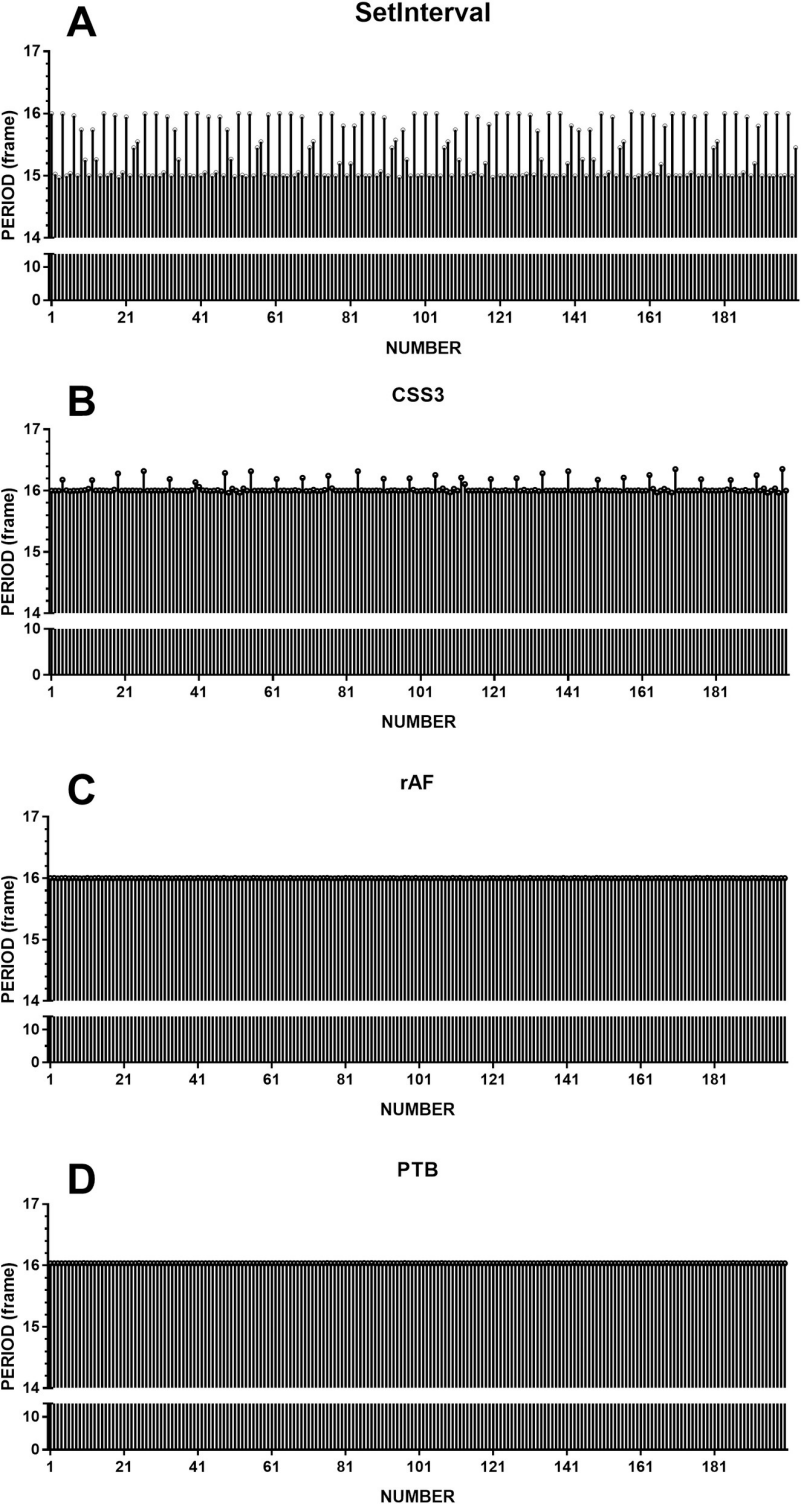
The time distribution of 200 sampling periods of dynamic sinusoidal gratings using different methods. A: the 200 periods produced by setInterval were randomly distributed between 14.9 frames and 16.1 frames; 150 frame losses occurred. B: the 200 periods produced by CSS3 were randomly distributed between 15.9 frames and 16.4 frames; 29 frame losses occurred. C: the 200 periods produced by rAF were 16.0 frames, and no frame losses occurred. D: the 200 periods produced by PTB were 16.0 frames, and no frame losses occurred. C and D show a similar flatness, which means the timing accuracy of rAF is consistent with PTB.

Fig 1 shows 200 periods that were extracted. The horizontal axis indicates the sequence number of periods; the vertical axis indicates the period. We can see that there are many frame losses in Fig 1A and 1B while the graphs in Fig 1C and 1D are fairly flat. Fig 1A, produced by setInterval, shows that the real periods (mean = 15.4 frames, range = 1.05 frames, SD = 0.43 frames, FLR = 75.0%) were always shorter than the intended periods (16 frames). Meanwhile, Fig 1B, produced by CSS3, shows longer periods (mean = 16.0 frames, range = 0.39 frames, SD = 0.09 frames, FLR = 14.5%). Apparently, the timing accuracy of the dynamic sinusoidal grating using rAF technology (mean = 16.0 frames, range = 0.007 frames, SD = 0.001 frames, FLR = 0.00%) on Chrome is consistent with that of PTB (mean = 16.0 frames, range = 0.003 frames, SD = 0.005 frames, FLR = 0.00%).

**Fig 2:**
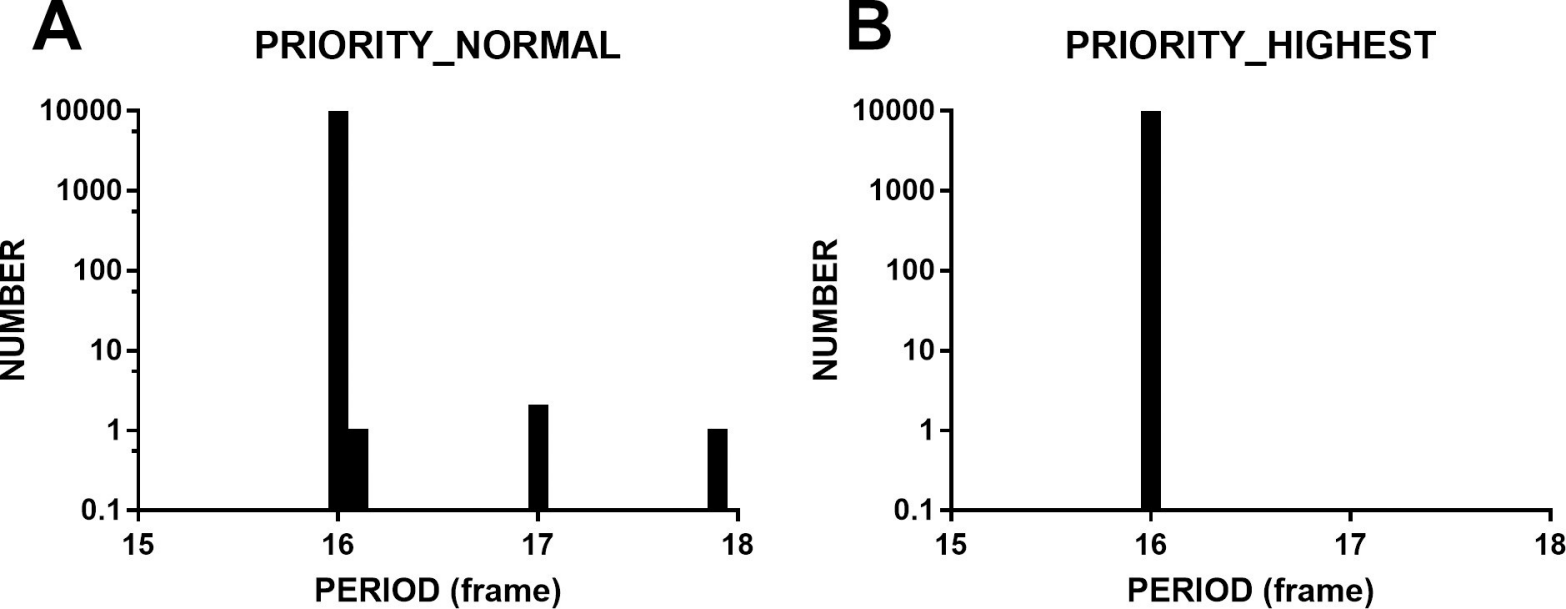
A: shows running at normal priority, and B: shows running at real-time priority. The periods at normal priority were distributed between 15.9 frames and 18.0 frames, and longer periods occurred rarely (mean = 16.0 frames, SD = 0.02 frames, range = 1.9 frames, FLR = 0.04%). The periods at real-time priority were 16.0 frames, and no longer periods occurred (mean = 16.0 frames, SD = 0.001 frames, range = 0.01 frames, FLR = 0%).

Two hundred periods were not long enough to satisfy real experiments. Therefore, we examined the timing accuracy of the dynamic sinusoidal grating with 10,000 periods using rAF technology. To clearly show the distribution of frames, a base-10 log-axis was applied to the y-axis. The results are shown in Fig 2A. The unit of the horizontal axis is in frames; the vertical ordinate is the number of grating stimuli in each bin (bin width = 0.1 frames). Several longer periods appeared. The Psychtoolbox experiment indicated that the priority of the stimulus would affect timing accuracy. Therefore, we manually boosted the priority of Chrome to real time when gratings were presented. The results are shown in Fig 2B. The longer periods disappeared, and the timing accuracy was highly improved (mean = 16.0 frames, SD = 0.001 frames, range = 0.01 frames, FLR = 0.0%), to the same level as PTB (mean = 16.0 frames, SD = 0.001 frames, range = 0.006 frames, FLR = 0.0%). The results that follow were measured at real-time priority unless otherwise stated.

**Fig 3:**
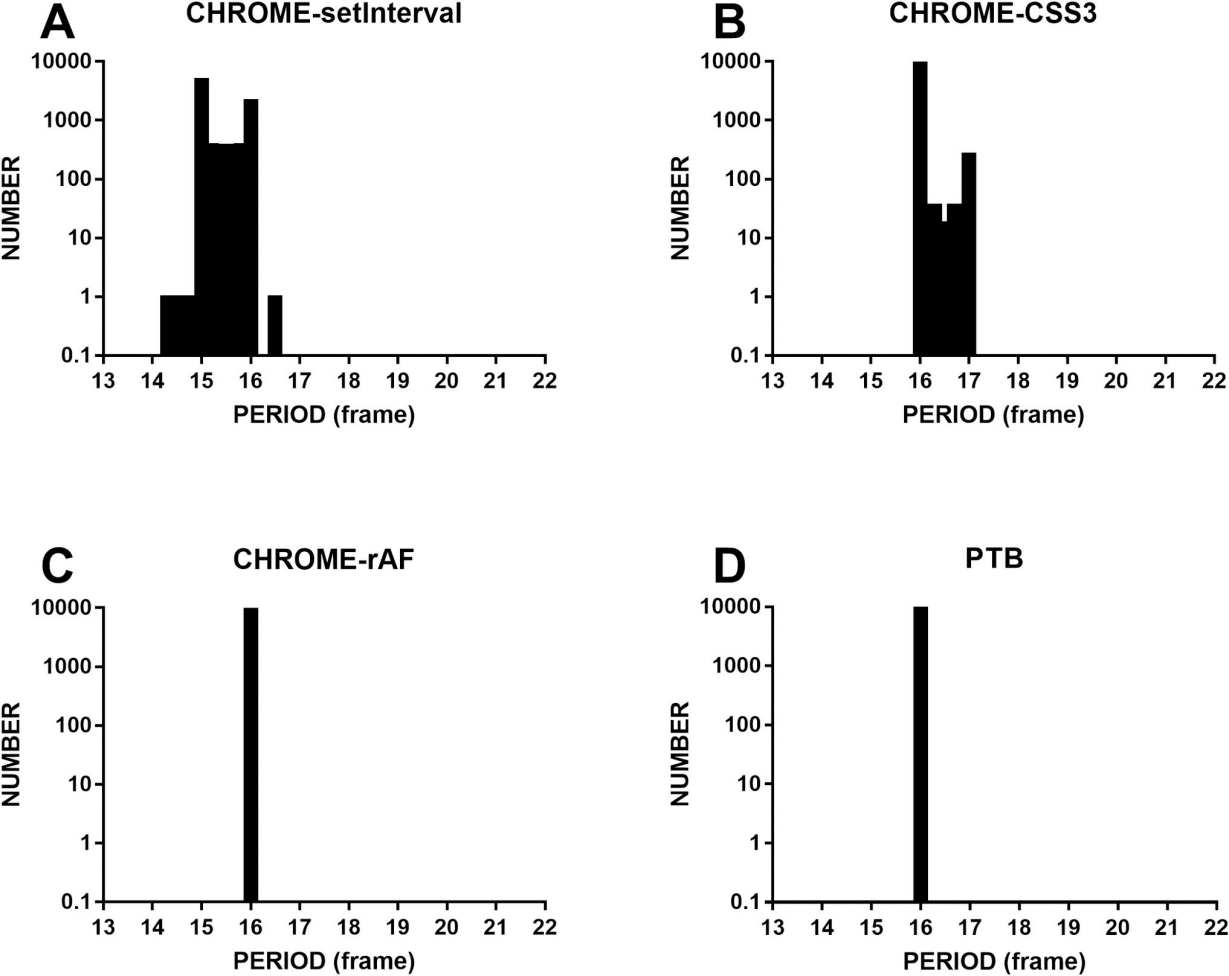
The four histograms show the different methods using Chrome and PTB to show 10,000 periodic distributions of grating stimuli. Bar height indicates the number of gratings distributed over the period. The ordinate is the logarithmic base 10 for showing small numbers. A: illustrates the periodic distribution of gratings designed by setInterval, and the distribution is relatively scattered. B: illustrates the periodic distribution of gratings designed by CSS3, and the distribution is also relatively scattered. C: illustrates the periodic distribution of gratings designed by rAF, and the distribution is concentrated at the 16th frame. D: illustrates the periodic distribution of gratings designed by PTB, and only one bar stands at the 16th frame. C and D: indicate that the timing accuracy produced by rAF is as good as that of PTB.

We reassessed the timing accuracy of the dynamic sinusoidal gratings with 10,000 periods at real-time priority. The experiments were conducted on computer 1. The refresh rate of the monitor was set to 60 Hz. Data in the four inset figures of Fig 3 were collected at real-time priority on Chrome and PTB. The 10,000-period distribution of setInterval, which is illustrated in Fig 3A, is relatively scattered, distributed between 14.2 frames and 16.4 frames (mean = 15.4 frames, range = 2.2 frames, SD = 0.4 frames, FLR = 75.7%). The distribution of CSS3, as shown in Fig 3B, is also scattered, distributed between 15.9 frames and 17.0 frames (mean = 16.0 frames, range = 1.1 frames, SD = 0.2 frames, FLR = 4.00%). Fig 3C shows the period distribution of gratings designed by rAF. The period is 16.0 frames (range = 0.01 frames, SD = 0.001 frames, FLR = 0.0%). Fig 3D measures gratings generated by PTB, which is used in traditional experiments; its period is 16.0 frames. Fig 3 indicates that the period distribution of rAF is more concentrated than that of setInterval and CSS3, and is almost the same as that of PTB. In number grade, the timing accuracies of rAF and PTB are consistent (rAF: mean = 16.0 frames, range = 0.01 frames, SD = 0.001 frames, FLR = 0.0%; PTB: mean = 16.0 frames, range = 0.01 frames, SD = 0.001 frames, FLR = 0.0%).

**Fig 4:**
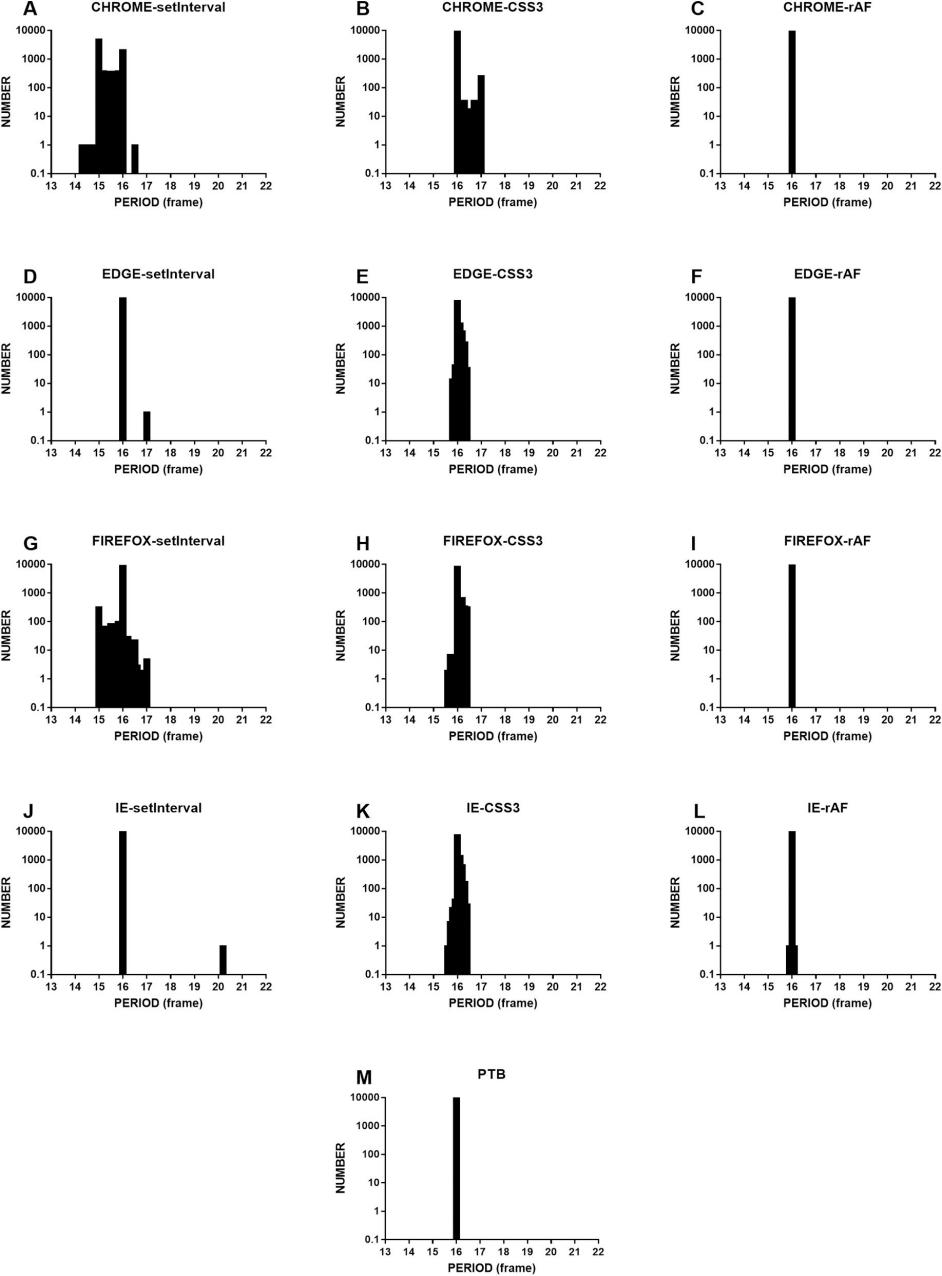
The histograms show the period data for grating experiments in Chrome, Firefox, Edge, and Internet Explorer (hereafter, IE) using setInterval, CSS3, and rAF. The bottom of the figure shows the PTB period as the gold standard. All data ran at real-time priority. The unit of the abscissa is frames (screen refresh rate is 60 Hz), and the ordinate indicates the number of grating appearances. The three figures in the first row show the results of the different methods tested in Chrome; the data are the same as that presented in Fig 3 but are displayed in a different coordinate system. The experimental results of the different methods tested in Edge, Firefox, and IE are also shown. The last row shows the experimental results from PTB; rAF showed the highest timing accuracy among the web technologies and was the closest to that of PTB.

Owing to the compatibility of programs with different browsers, we also experimented in Edge, Internet Explorer, and Firefox. The sampling rate of the logic analyzer was set to 1.6 MS/sec for the digital signal. All indicated that the timing accuracy of rAF was consistent with that of PTB and was much higher than those of setInterval and CSS3 (Fig 4).

**Fig 5:**
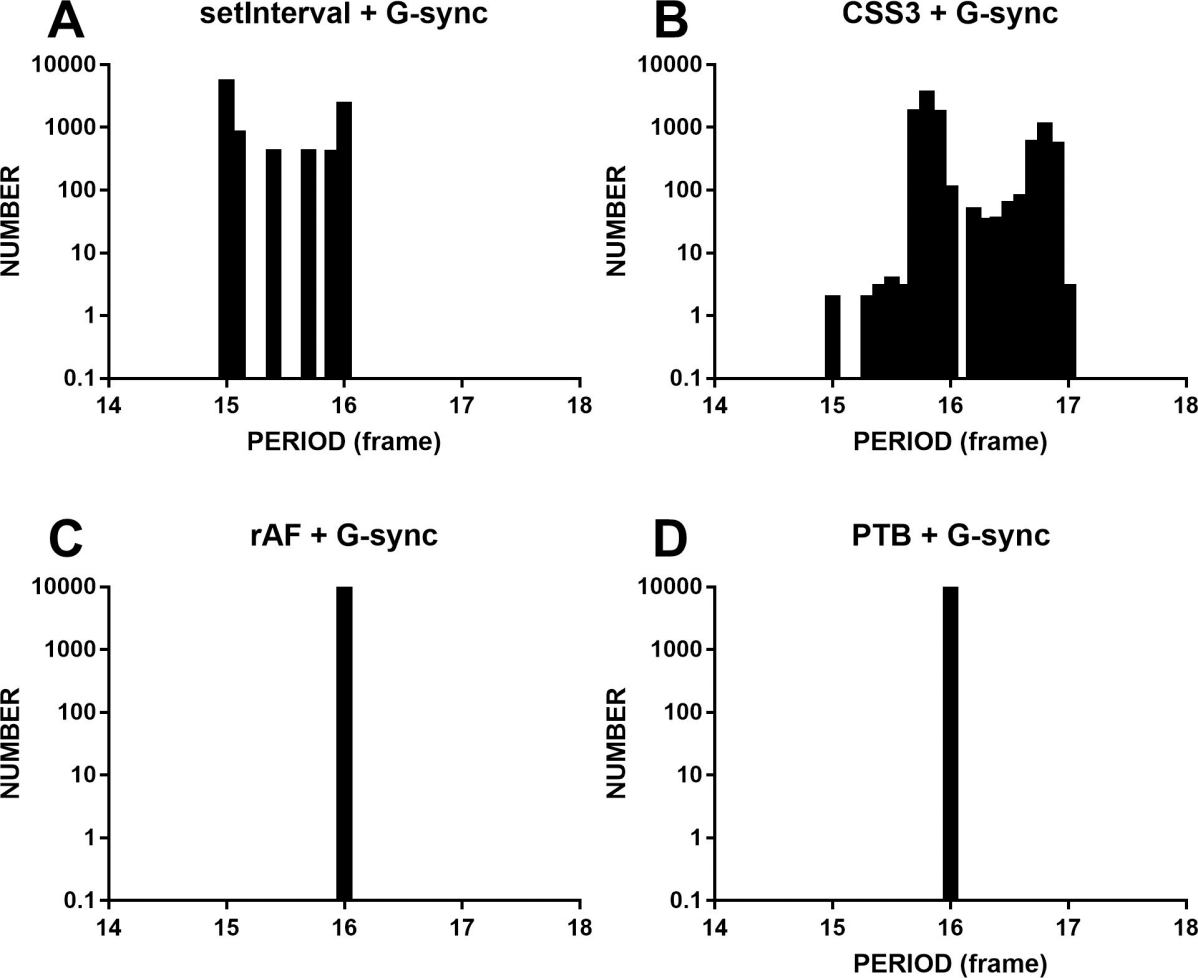
The four histograms are the periodic distributions of grating stimuli for the different methods in Chrome and PTB. The computer was powered by G-sync technology, and the refresh rate was 60 Hz. The titles of the subplots indicate the methods for producing the grating. The scattering of the bars in A and B indicates that G-sync did not improve the timing accuracies of setInterval and CSS3. The one bar in C and D indicates that the timing accuracies of rAF and PTB also did not benefit from G-sync.

G-sync is designed to smooth out gameplay and prevent screen tearing. Here, we tested whether G-sync technology could also improve the timing accuracy of the dynamic sinusoidal grating designed by setInterval or CSS3. This experiment was conducted on computer 2 (Chrome, Windows 10, 60 Hz). G-sync was enabled in the NVIDIA control panel. The refresh rate was set to 60 Hz. Results are presented in Fig 5. Fig 5A and 5B show broad distribution in frames 15–17 while rAF and PTB show a very narrow distribution. Fig 5 indicates that the timing accuracy of web technologies could not benefit from G-sync technology, except at higher refresh rates (here, we must note that Edge and IE do not support 144 Hz, even when G-sync is enabled).

**Fig 6:**
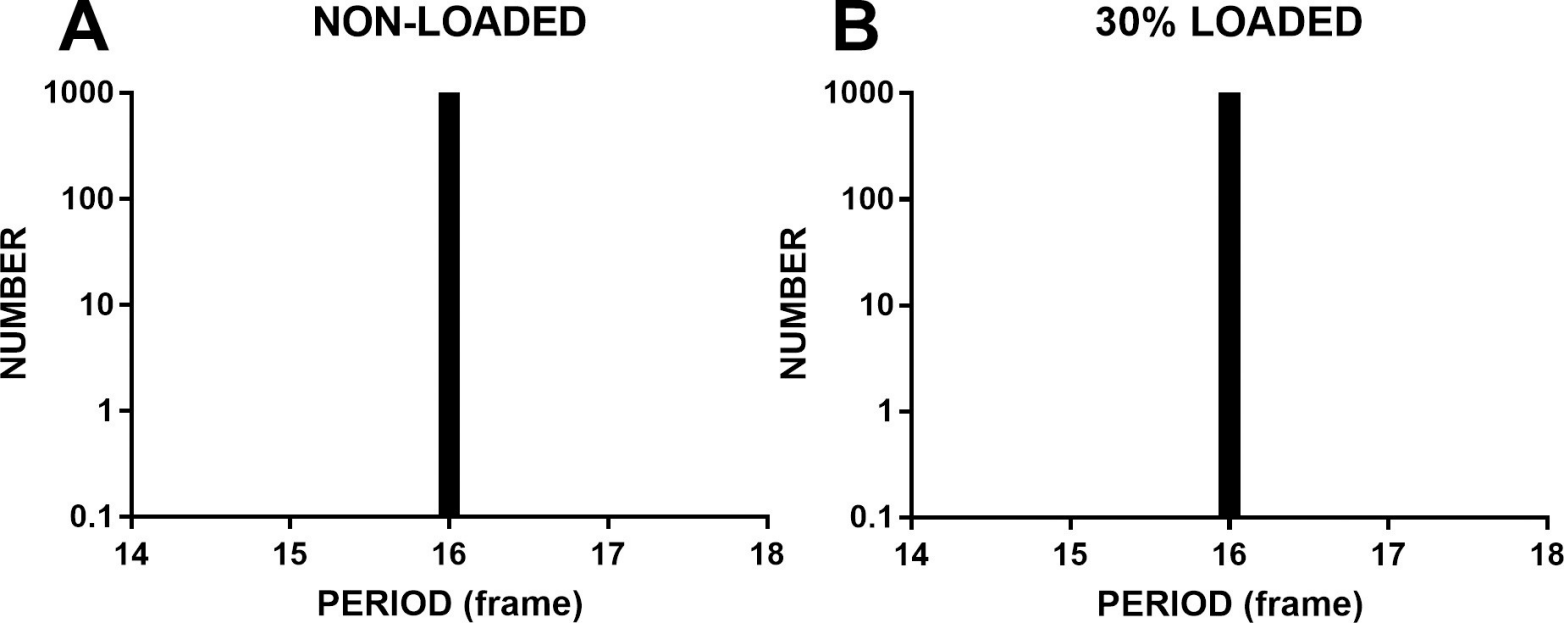
The timing accuracy of the dynamic sinusoidal grating designed by rAF run at 30% of the CPU utilization rate. The priority was set to the real-time level, and the refresh rate was 60 Hz. There is only one bar standing at the 16th frame, which indicates that the timing accuracy (mean = 16.0 frames, SD = 0.001 frames, range = 0.004 frames, FLR = 0.00%) was very high at 30% of the CPU utilization rate.

A higher CPU utilization rate might affect the performance of browsers and thus affect timing accuracy. To test this, we ran a dynamic sinusoidal grating designed by rAF in Chrome at 30% of CPU utilization rate on computer 2. When the grating stimulus was presented, a MATLAB routine was running to find rising edges of a periodic wave. The MATLAB routine raised the CPU utilization to 30%. The priority was set at the real-time level. Results are shown in Fig 6. Fig 6B clearly shows that the dynamic sinusoidal grating drifted smoothly and maintained high timing accuracy (mean = 16.0 frames, SD = 0.001 frames, range = 0.004 frames, FLR = 0.00%).

**Fig 7:**
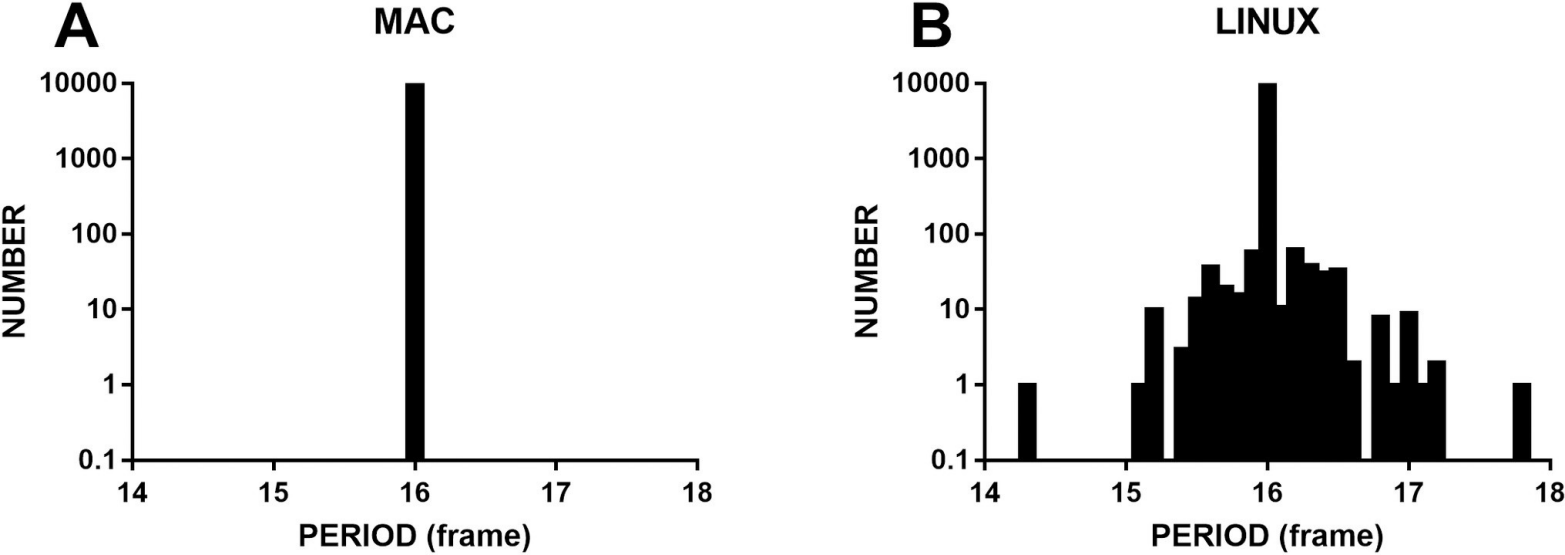
The timing accuracy of the dynamic sinusoidal grating designed by rAF in macOS and Linux. The priority was set to the real-time level, and the refresh rate was 60 Hz. A shows that only one bar is standing at the 16th frame, which indicates that the accuracy of the grating could reach high precision (mean = 16.0 frames, SD = 0.0004 frames, range = 0.003 frames, FLR = 0.00%). However, B shows that the grating had poor timing accuracy (mean = 16.0 frames, SD = 0.08 frames, range = 3.5 frames, FLR = 3.4%) in Linux.

To check whether rAF would also have the highest time accuracy in other operating systems, we ran the same procedures in Chrome on macOS (High Sierra 10.13.6) with default priority and Linux (ubuntu 19.04, computer 2) with real-time priority. The refresh rate was set to 60 Hz. On macOS (High Sierra 10.13.6), the dynamic grating ran smoothly with high timing accuracy (mean = 16.0 frames, SD = 0.0004 frames, range = 0.003 frames, FLR = 0.00%); see Fig 7A. However, rAF in Chrome on Linux showed poor timing accuracy (mean = 16.0 frames, SD = 0.08 frames, range = 3.5 frames, FLR = 3.4%); this is observed Fig 7B. It should be noted here that macOS ran on an older computer (MacBook pro, 15-inch, mid-2010).

### Experiment 2

Flashes are also popular stimuli used in psychology experiments. Their luminance change sharply, unlike dynamic sinusoidal gratings. Here, we measured the timing accuracy in a similar manner to the gratings. The flashes were run in Chrome, Firefox, Edge, and IE browsers on computer 1. White and black squares of 512 x 512 pixels were alternately drawn on a canvas in HTML5. The flash procedures can be found in the S1 File. Priority was set at the real-time level, and the refresh rate was 60 Hz. The temporal frequencies of the flashes were 30 Hz (2 frames/period) and 3.75 Hz (16 frames/period).

The results are presented in Table 1. As can be seen, there was a lot of frame loss in the tests using setInterval and CSS3. The results for rAF still showed high time accuracy, but longer or shorter periods occurred rarely—one or two frame losses in 10,000. The frame losses in the flash experiments resulted in a lower SD but a larger range. After excluding longer and shorter periods, the timing accuracy of the flashes designed by rAF demonstrated higher precision (ranges decreased to 0.01 frames in number grade), which is much closer to that of PTB. Therefore, setInterval and CSS3 cannot be reliably used to generate online experiments with precise stimulus presentations. Meanwhile, rAF can provide higher timing accuracies for stimulus presentations in most cases.

**Table 1:** The timing accuracies of flashes designed by PTB, setInterval, CSS3, and rAF. The monitor ran at 60 Hz. The temporal frequencies of flashes were 30 Hz (2 frames/period) and 3.75 Hz (16 frames/period). All flashes ran at real-time priority.

Period (frames)	PTB and Browser	Technology	Mean	SD	Range	Longer periods	Shorter periods	FLR
	PTB		2.0	0.0011	0.01	0	0	0.00%
30 Hz (2 Frames)	Chrome	setInterval	2.1	0.30	2.21	870	191	10.6%
		CSS3	2.1	0.59	15.13	348	32	3.80%
		rAF	2.0	0.0097	0.99	1	0	0.01%
	Edge	setInterval	2.0	0.046	1.26	19	2	0.21%
		CSS3	2.1	0.52	6.26	194	8	2.02%
		rAF	2.0	0.0067	0.67	0	1	0.01%
	Firefox	setInterval	3.3	0.62	5.23	9682	70	97.52%
		CSS3	2.0	0.044	1.25	18	5	0.23%
		rAF	2.0	0.0013	0.01	0	0	0.00%
	IE	setInterval	2.0	0.016	0.16	0	0	0.00%
		CSS3	2.1	0.51	6.25	193	15	2.08%
		rAF	2.0	0.0008	0.05	0	0	0.00%
	PTB		16.0	0.0004	0.003	0	0	0.00%
3.75 Hz (16 Frames)	Chrome	setInterval	16.0	0.61	14.1	427	861	12.88%
		CSS3	16.0	0.18	2.00	170	162	3.32%
		rAF	16.0	0.014	1.00	2	0	0.02%
	Edge	setInterval	16.0	0.42	3.00	673	1067	17.40%
		CSS3	16.0	0.16	2.01	133	119	2.52%
		rAF	16.0	0.014	2.00	1	1	0.02%
	Firefox	setInterval	16.9	0.37	9.81	8748	7	87.55%
		CSS3	16.0	0.052	2.00	22	5	0.27%
		rAF	16.0	0.01	1.00	1	0	0.01%
	IE	setInterval	16.0	0.52	13.3	667	1091	17.58%
		CSS3	16.0	0.16	2.01	135	123	2.58%
		rAF	16.0	0.014	2.00	1	1	0.02%

### Experiment 3

**Fig 8:**
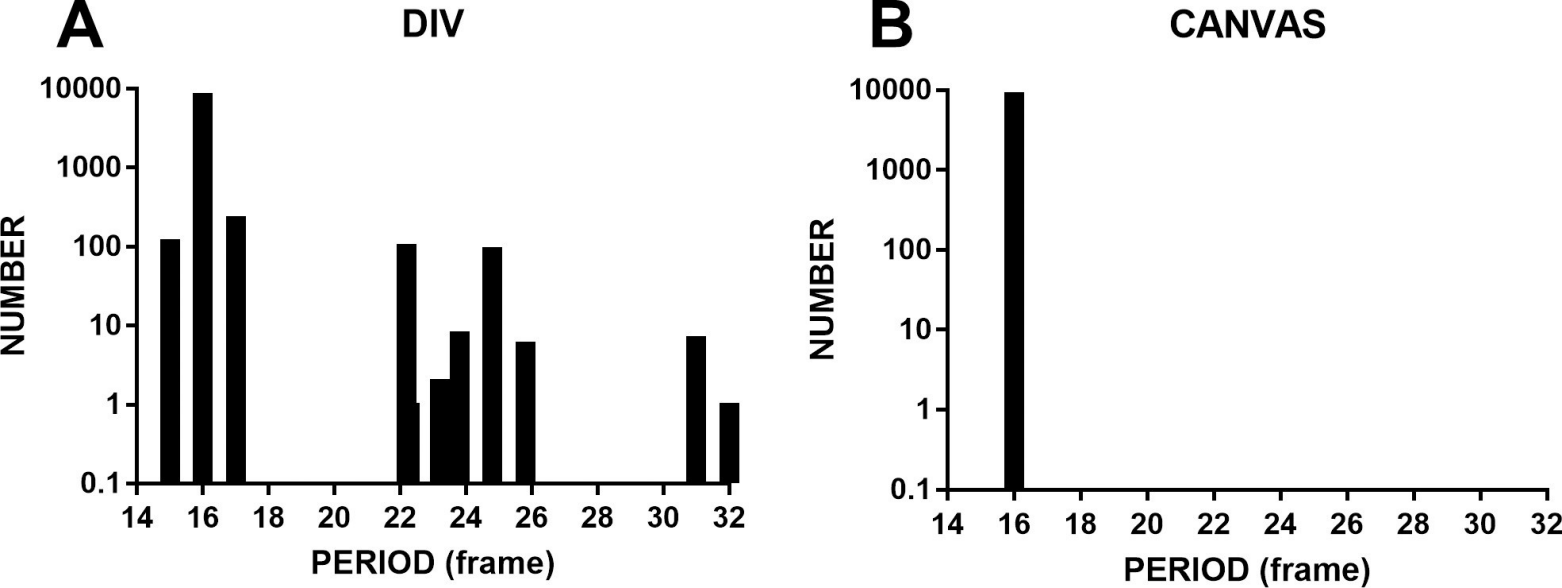
The timing accuracies of flashes produced by rAF in div tags and on a canvas. A shows a broader distribution of periods, ranging from 15.0 to 32.0 frames. B shows an extremely narrow distribution (mean = 16.0 frames, SD = 0.002 frames, range = 0.007 frames, FLR = 0.0%); only one bar stands at the 16th frame. The results show that presenting stimuli on a canvas is essential for getting precise timing.

There are two methods for producing flash stimuli using rAF technology. One is changing the color of the div tag, and the other is drawing alternating white and black squares on a canvas (a tag of HTML5). Here, we tested the accuracy of the timing of these two methods at a frame rate of 144 Hz. This experiment was conducted on computer 2 (Chrome, Windows 10). G-sync was enabled in the NVIDIA control panel. The temporal frequency of the flash was set to 11.2 Hz (16 frames/period). The results showed that the flash displayed on the canvas had more accurate timing (Fig 8B, mean = 16.0 frames, SD = 0.002 frames, range = 0.007 frames, FLR = 0.0%). The accuracy of the timing of the first method showed a wider distribution than the second method, ranging from 15.0 frames to 32.0 frames. This result indicates that presenting stimuli on a canvas is essential for achieving precise timing.

### Experiment 4

The duration of a CSS3 animation must be set to an exact time while the refresh interval includes an infinite decimal, such as 1/60 seconds. When an infinite decimal is truncated to adapt to CSS3 syntax, residual errors will be accumulated frame by frame. For example, the exact time of two frames is 1000/30 ms. If 33.3 ms is written, an error of 100 / 3–33.3 ≈ 0.033 ms will be generated, and this error is accumulated frame by frame. To know the effect of the accumulated error, we measured the results of the flash experiment with a period of six frames, in which the parameter of time could be set exactly to 100 ms (refresh rate = 60 Hz). This experiment was conducted on computer 1 (Chrome, Windows 10). Results showed that the range was as big as 6.20 frames, and the frame loss rate was as high as 4.9%. Therefore, the timing inaccuracy of CSS3 might not be sourced from the truncation of infinite decimals.

### Experiment 5

When browsers ran at real-time priority, the keyboard and mouse were not blocked, which is different from when Psychtoolbox is run. Keyboard and mouse events might disturb the presentation of dynamic stimuli. To evaluate the effect of keyboard and mouse interference on timing accuracy, we continuously tapped the keyboard and mouse with random intervals of 0.5–5 s, when the dynamic sinusoidal gratings for 1000 cycles (approximately 4 min and 45 s) were presented. This experiment was conducted on computer 2 (Chrome, Windows 10, 60 Hz). The timing accuracy was as follows: mean = 16 frames, SD = 5.74 × 10^-4^ frames, range = 0.004 frames, and frame loss rate = 0.0%. These results are almost the same as those presented in Fig 3C.

## Discussion

In a web browser, the timing accuracy of rAF is much closer to that of PTB and much more accurate than that of other web technologies, and it can be improved by boosting the priority. rAF is also very compatible with different browsers. It could serve as a potential technology for online experiments on Windows and macOS, but not on Linux. Therefore, rAF can solve the problem of low timing accuracy in web browsers while also addressing the issues raised by Plant [5].

Garaizar and Peips (2018) suggested that rAF shows high timing accuracy in operating systems except those based in Linux [11]. In the present study, we obtained similar results using the typical stimuli of dynamic sinusoidal gratings and flashes. However, they did not report the frame loss in some of their results on Linux could have been attributable to the only hundreds of periods measured.

Here, we tried to clarify why rAF could achieve millisecond timing accuracy in web browsers. First, we should note the difference between the screen refresh rate and the frame rate. Frame rate is the frequency at which a GPU renders the screen, and the screen refresh rate refers to the liquid crystal display’s refresh rate. The screen refresh rate follows changes in the display screen [12,13], and the screen refresh rate of most displays is 60 Hz. A web page is drawn by graphical processing unit (GPU) or CPU, and the frequency of drawing is limited by the screen refresh rate. The rAF method’s rendering time follows the screen refresh rate. If the screen refresh rate is 1/60 ms, it will be drawn in 1/60 ms. The internal rendering principle of rAF applies for a new frame, and it can run the callback function at the same time. In addition, the rAF approach is almost the same as that of Psychtoolbox [10,14]. Hence, it is not surprising that rAF can achieve millisecond timing accuracy.

**Fig 9:**
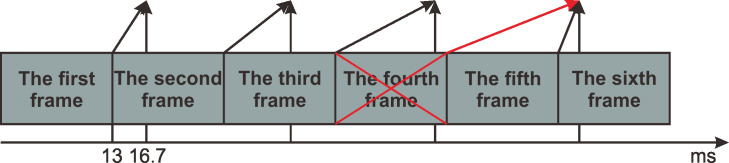
Frame rendering mismatched with the screen refresh rate. Suppose the screen refresh rate is 60 Hz (refresh period is approximately 16.7 ms), and the screen rendering period is set to 13 ms. Since the browser renders a page every 16.7 ms, the browser refreshes the previous picture when the page finishes rendering the next frame. Then, frame 4 becomes lost.

What will happen if we use web technologies where the frame rate can be set to not match the screen refresh rate [15]? If the screen refresh rate is 60 Hz (i.e., the refresh period is approximately 16.7 ms), and we set the frame rate to 13 ms (see Fig 9), because the browser renders a page every 16.7 ms, the browser will refresh the previous picture when the page rendered the next frame. This could cause the fourth frame to be lost in the page, and so on. This would periodically produce frame loss.

The Web Animation API mentioned in the introduction inherits the performance of CSS3 and the flexibility of JavaScript [16]. However, the principle of Web Animation API is similar to that of CSS3 and therefore might not be the right new technology for achieving high timing accuracy in experiments.

This study has some limitations. First, we measured timing accuracy in specific browser versions. It should be noted that results should be consistent across operation systems according to the HTML5 standard. In fact, even rAF performs very differently on different browsers in Linux. Therefore, there is no assurance of timing accuracy in a new version of a specific browser. Second, we did not measure the timing accuracy of complex or natural stimuli due to technological limitations. There should have been more consideration of timing accuracy when using complex stimuli. Timeline tools in Chrome might be helpful for assessing the timing accuracy of complex stimuli. Third, rAF only improved the timing accuracy of dynamic stimuli, which are only a part of the timing accuracy of the whole system [17]. For example, the accuracy of button detection should be carefully considered in response-time-related experiments. Finally, rAF was not perfect in all experiments, and more consideration should be taken in future experiments related to highly accurate timing.

Browser-based experiments are expected to be generalizable to the Internet. However, care should be taken for specific applications in terms of timing accuracy. If data collection requires high timing accuracy, users need to boost the web browser to real-time priority. In that case, we should provide good introductions (e.g., videos or animations) to guide participants on how to boost priority.

## Supporting information

S1 File (ZIP)

## References

[1] Dandurand F, Shultz TR, Onishi KH (2008) Comparing online and lab methods in a problem-solving experiment. Behav Res Methods 40: 428–434. 10.3758/brm.40.2.428 18522052

[2] Henninger F, Shevchenko Y, Mertens UK, Kieslich PJ, Hilbig BE. lab.js: A free, open, online study builder. PsyArXiv; 2019 10.31234/osf.io/fqr49 PMC9046347 34322854

[3] de Leeuw JR (2015) jsPsych: a JavaScript library for creating behavioral experiments in a Web browser. Behav Res Methods 47: 1–12. 10.3758/s13428-014-0458-y 24683129

[4] de Leeuw JR, Motz BA (2016) Psychophysics in a Web browser? Comparing response times collected with JavaScript and Psychophysics Toolbox in a visual search task. Behav Res Methods 48: 1–12. 10.3758/s13428-015-0567-2 25761390

[5] Plant RR (2016) A reminder on millisecond timing accuracy and potential replication failure in computer-based psychology experiments: An open letter. Behav Res Methods 48: 408–411. 10.3758/s13428-015-0577-0 25761394

[6] Schmidt WC (2001) Presentation accuracy of Web animation methods. Behav Res Methods Instrum Comput 33: 187–200. 10.3758/bf03195365 11447672

[7] van Steenbergen H, Bocanegra BR (2016) Promises and pitfalls of Web-based experimentation in the advance of replicable psychological science: A reply to Plant (2015). Behav Res Methods 48: 1713–1717. 10.3758/s13428-015-0677-x 26542973 PMC5101252

[8] Reimers S, Stewart N (2007) Adobe Flash as a medium for online experimentation: a test of reaction time measurement capabilities. Behav Res Methods 39: 365–370. 10.3758/bf03193004 17958146

[9] Web Animations. https://www.w3.org/TR/web-animations-1/. 2018.

[10] Brainard David H (1997) The Psychophysics Toolbox. Spat Vis 10: 433–436. 9176952

[11] Garaizar P, Reips UD (2018) Best practices: Two Web-browser-based methods for stimulus presentation in behavioral experiments with high-resolution timing requirements. Behav Res Methods. 10.3758/s13428-018-1126-4 30276629

[12] Lagroix HEP, Yanko MR, Spalek TM (2012) LCDs are better: psychophysical and photometric estimates of the temporal characteristics of CRT and LCD monitors. Atten Percept Psychophys 74: 1033–1041. 10.3758/s13414-012-0281-4 22359147

[13] Plant RR, Turner G (2009) Millisecond precision psychological research in a world of commodity computers: new hardware, new problems? Behav Res Methods 41: 598–614. 10.3758/BRM.41.3.598 19587169

[14] Pelli Denis G (1997) The VideoToolbox software for visual psychophysics: transforming numbers into movies. Spat Vis 10: 437–442. 9176953

[15] Woods AT, Velasco C, Levitan CA, Wan X, Spence C (2015) Conducting perception research over the internet: a tutorial review. PeerJ 3: e1058 10.7717/peerj.1058 26244107 PMC4517966

[16] Using the Web Animations API. https://developer.mozilla.org/en-US/docs/Web/API/Web_Animations_API/Using_the_Web_Animations_API. 2018.

[17] Reimers S, Stewart N (2015) Presentation and response timing accuracy in Adobe Flash and HTML5/JavaScript Web experiments. Behav Res Methods 47: 309–327. 10.3758/s13428-014-0471-1 24903687 PMC4427652

