# Supplementary material for: Implementation of stimuli with millisecond timing accuracy in online experiments
Source: PLoS One. 2020 Jul 10;15(7):e0235249. doi: 10.1371/journal.pone.0235249 (PMC7351209; doi:10.1371/journal.pone.0235249)
Supplement: S1 File — (ZIP) [file pone.0235249.s001.zip › Supplemental files/readme.pdf]

procedures

procedures folder: the procedures to generate dynamic sinusoidal grating and flash.

"flash-CSS3": The procedure to generate flash using CSS3 in canvas tag.
"flash-rAF-canvas": The procedure to generate flash using rAF in canvas tag.
"flash-rAF-div": The procedure to generate flash using rAF in div tag.
"flash-setinterval": The procedure to generate flash using setInterval in canvas tag.
"grating-CSS3": The dynamic sinusoidal grating was generated using CSS3 in canvas tag.
"grating-rAF": The dynamic sinusoidal grating was generated using rAF in canvas tag.
"grating-setinterval": The dynamic sinusoidal grating was generated using setInterval in canvas tag.
"flashPTB.m": The procedure to generate flash using Psychtoolbox.
"gratingPTB.m": The dynamic sinusoidal grating was generated using Psychtoolbox.

data

data folder: the folder includes the middle-results

"./data/Flash/flash highest 60Hz period 16 frames LINUX.csv": The data of Flash experiment in highest priority, and the period was set to 16 frames in LINUX. The monitor ran at 60 Hz.
"./data/Flash/flash realtime 60Hz period 2 frames.csv": The data of Flash experiment in realtime priority, and the period was set to 2 frames. The monitor ran at 60 Hz.
"./data/Flash/flash realtime 60Hz period 16 frames.csv": The data of Flash experiment in realtime priority, and the period was set to 16 frames. The monitor ran at 60 Hz.
"./data/Flash/flash realtime 144Hz period 16 frames with G-sync rAF.csv": The data of Flash experiment in realtime priority, and the period was set to 16 frames. The monitor ran at 144 Hz. G-sync was enabled.
"./data/Flash/flash realtime 144Hz period 16 frames without G-sync rAF.csv": The data of Flash experiment in realtime priority, and the period was set to 16 frames. The monitor ran at 144 Hz.

"./data/Grating/grating different priority 60Hz.csv": The data of Grating experiment in different priority, and the period was set to 16 frames. The monitor ran at 60 Hz.
"./data/Grating/grating realtime 60Hz diferent OS.csv": The data of Grating experiment in realtime priority at different OS, and the period was set to 16 frames. The monitor ran at 60 Hz.
"./data/Grating/grating realtime 60Hz different methods.csv": The data of Grating experiment in realtime priority at different methods, and the period was set to 16 frames. The monitor ran at 60 Hz.
"./data/Grating/grating realtime 60Hz G-sync.csv": The data of Grating experiment in realtime priority, and the period was set to 16 frames. The monitor ran at 60 Hz. G-sync was enabled.
"./data/Grating/grating realtime 60Hz Loaded.csv": The data of Grating experiment in realtime priority with 30% loaded, and the period was set to 16 frames. The monitor ran at 60 Hz.
"./data/Grating/grating realtime 60Hz.csv": The data of Grating experiment in realtime priority, and the period was set to 16 frames. The monitor ran at 60 Hz.
"./data/Flash supplement table.csv": Table 1: The statistics of Flash experiment in realtime priority, and the period was set to 16 frames. The monitor ran at 144 Hz. Table 2: The statistics of Flash experiment in highest priority at LINUX, and the period was set to 16 frames. The monitor ran at 60 Hz.

example

an example of online visual experiments

The experiment in Landau & Fries (2012) was transplanted to the Web browser.

phototriode circuit

"phototriode circuit.pdf": the circuit of photo-electric convertor used in our measure-system. you can easily have it by amozon.com (e.g. https://www.amazon.com/DROK-Detection-Photosensitive-Photodiode-Directional/dp/B00IM8OD14/ref=sr_1_8?keywords=Photosensitive+Sensor+Module&qid=1560223954&s=gateway&sr=8-8#customerReviews)
"logic analyzer.jpg": a photograph of our setup with the logic analyzer.
