# Supplementary figures and images for: Implementation of stimuli with millisecond timing accuracy in online experiments
Source: PLoS One. 2020 Jul 10;15(7):e0235249. doi: 10.1371/journal.pone.0235249 (PMC7351209; doi:10.1371/journal.pone.0235249)

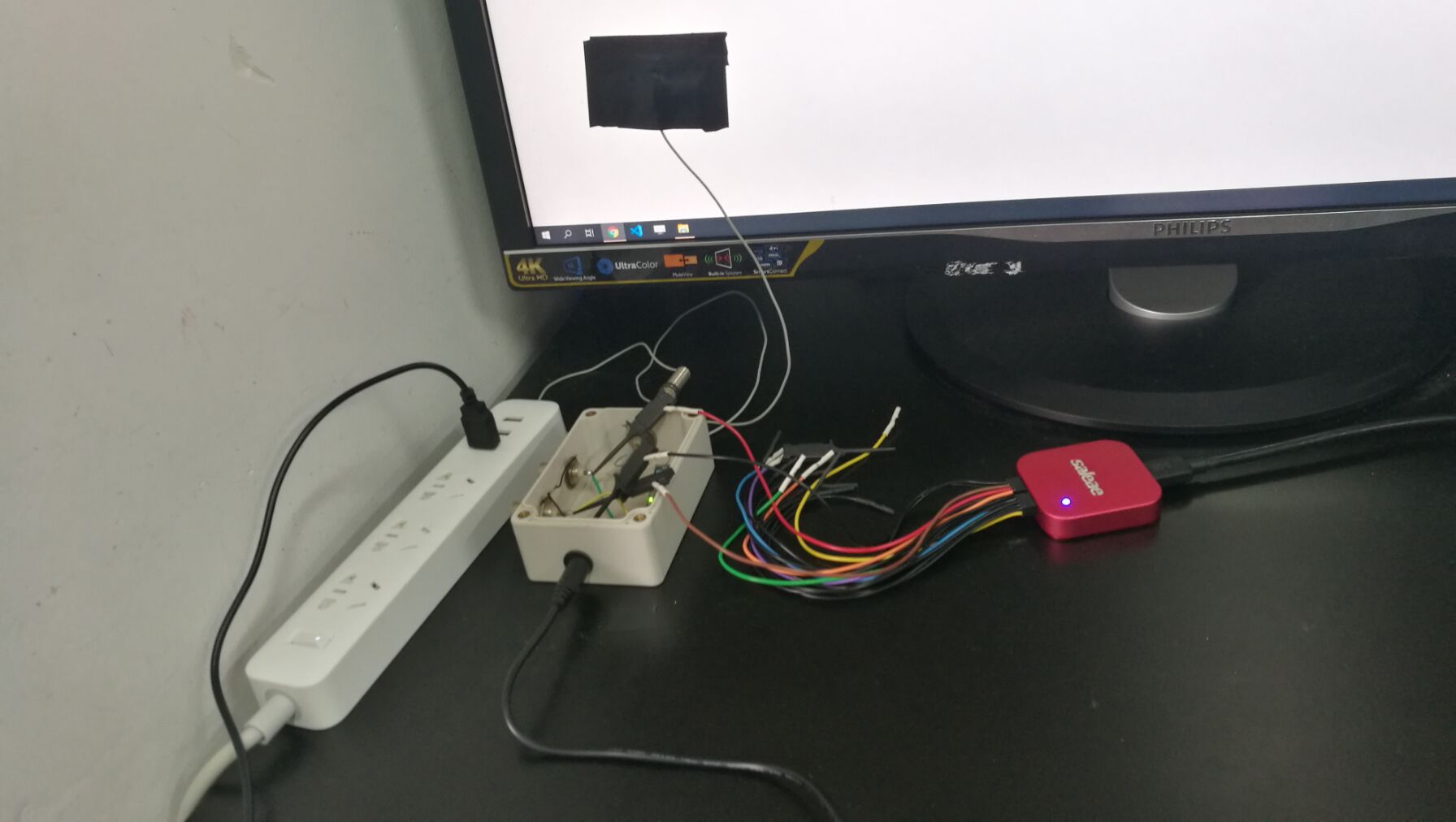

Supplement: S1 File — (ZIP) [file pone.0235249.s001.zip › Supplemental files/logic analyzer.jpg]

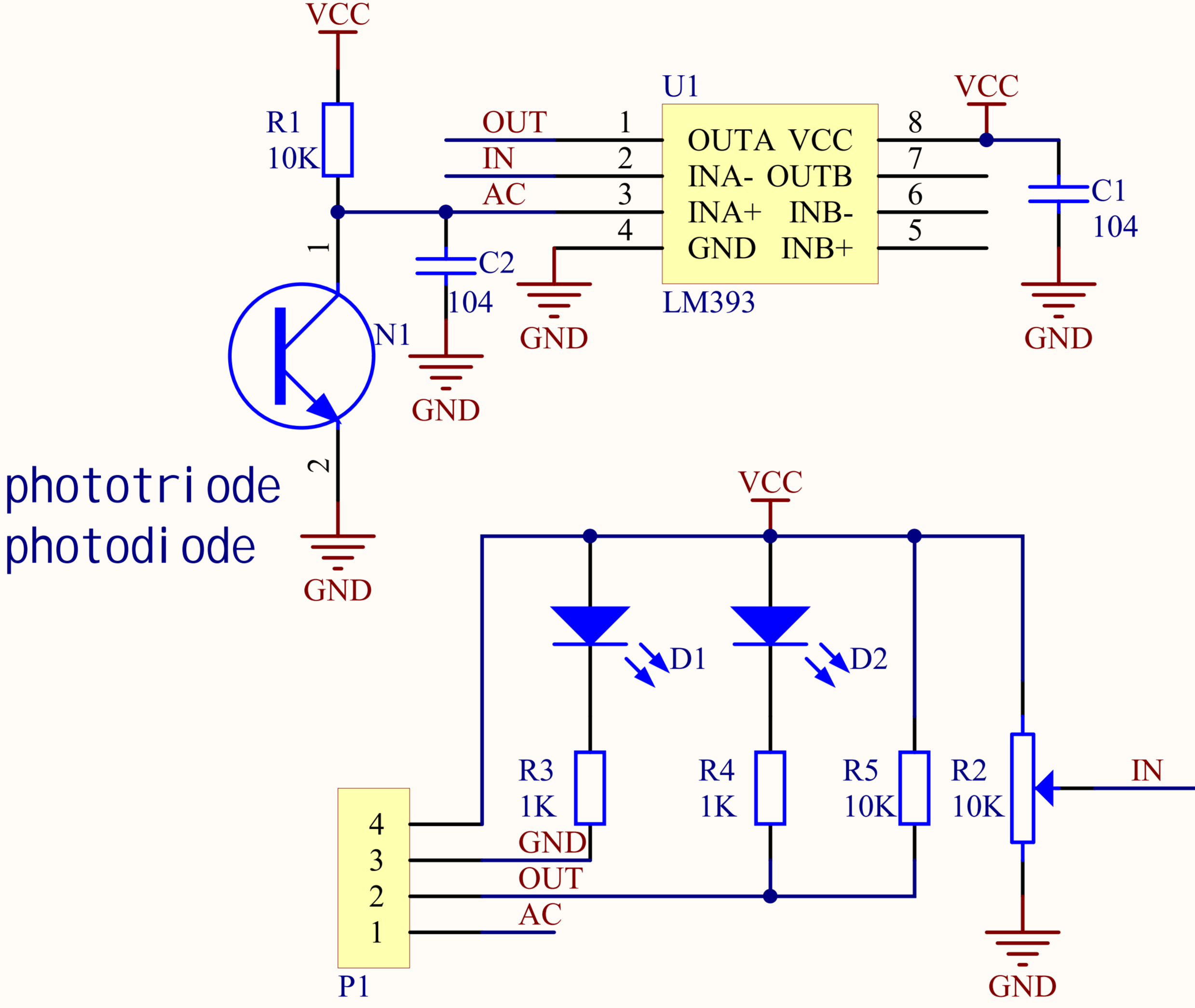

Supplement: S1 File — (ZIP) [file pone.0235249.s001.zip › Supplemental files/phototriode circuit.pdf]
